# CFD Simulation Study on Nitrogen Injection to Suppress Coal Spontaneous Combustion in Goaf under Downward-Ventilation Conditions

**DOI:** 10.1021/acsomega.5c02444

**Published:** 2025-06-20

**Authors:** Jianwei Wang, Xianfeng Liu

**Affiliations:** † Mining Disaster Prevention and Control-Ministry of State Key Laboratory Breeding Base, College of Safety and Environmental Engineering, Shandong University of Science and Technology, Qingdao 266590, China; ‡ State Key Laboratory of Coal Mine Disaster Prevention and Control, CCTEG Chongqing Research Institute, Chongqing 400037, China; § State Key Laboratory of Coal Mine Disaster Dynamics and Control, School of Resources and Safety Engineering, Chongqing University, Chongqing 400044, China

## Abstract

In the coal mining process, injecting nitrogen into the goaf is an effective method to suppress coal spontaneous combustion, which is widely adopted due to its significant inhibitory effect and low cost. Optimizing the injection volume and position of nitrogen is crucial for enhancing fire prevention and extinguishing efficiency and reducing safety risks. This study utilized CFD numerical simulation technology to investigate the spontaneous combustion risk in the goaf of the 81203 working face in Baode Coal Mine under the combined U + L ventilation and downwind ventilation conditions. The influence of different nitrogen injection positions and volumes on the distribution of the “three zones” related to coal spontaneous combustion in the goaf was analyzed. By simulating the changes in the oxygen and methane concentration fields in the goaf, this study proposed an optimized nitrogen injection strategy to improve fire prevention and extinguishing efficiency. The results show that setting a nitrogen injection port 90 m from the working face on the intake side of the 81203 auxiliary transport drift and injecting at a rate of 400m^3^/h meters per hour can achieve the best inhibitory effect and reduce costs.

## Graphical abstract

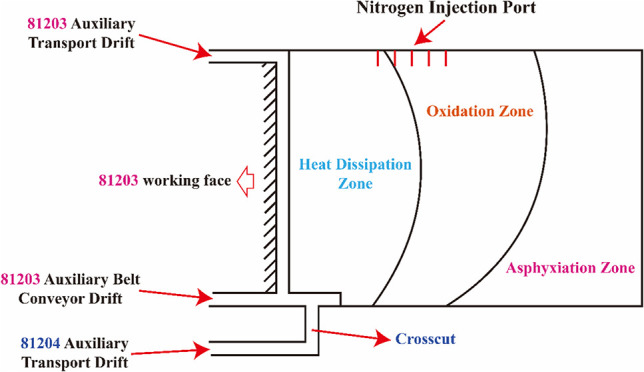

## 1 Introduction

The spontaneous combustion of residual coal in the goaf is one of the most significant disasters in coal mining production and a major challenge to coal mine safety. Bhattacharyya et al.^1^ proposed through their research that coal spontaneous combustion is primarily influenced by two factors: intrinsic factors (coal seam properties and geological conditions) and external factors (mining environment). Fires and explosions in coal mines are the most common and severe types of accidents, accounting for the highest proportion of major mine disasters. These incidents result not only in substantial property losses but, in more severe cases, in significant casualties.^2–6^ As a result, numerous scholars and technical experts have conducted extensive research on fire prevention and suppression technologies for the goaf in fully mechanized mining faces. Various methods, such as nitrogen injection, grouting, inhibitor spraying, and pressure equilibrium ventilation, have been proposed to control and prevent the spontaneous combustion of residual coal in the goaf.^7–16^

Nitrogen injection, as a critical measure for controlling spontaneous combustion in the goaf, effectively inertizes the goaf by rapidly displacing the air within, thereby reducing oxygen concentration and suppressing coal spontaneous combustion. Additionally, this method does not disrupt mining operations. In the event of a coal self-ignition incident, continuous nitrogen injection into the goaf can achieve effective fire suppression, significantly mitigating accidents caused by the spontaneous combustion of residual coal in the goaf.^17–19^

Wang Wenge et al.^20^ in addressing the issue of increased air leakage from high-extraction tunnels leading to a higher tendency for goaf spontaneous combustion, used Fluent software to simulate nitrogen injection in the goaf. They determined the distribution range of the spontaneous combustion “three zones” in the goaf and improved the high-extraction fire prevention and suppression system under nitrogen injection conditions. Wu Yuguo et al.^21^ injected nitrogen into the goaf using methods such as buried pipes and continuous nitrogen injection to prevent spontaneous combustion of residual coal in the goaf. They studied the distribution changes of the “three zones” of coal self-ignition in the goaf, with results indicating that under continuous nitrogen injection, the O_2_ concentration decreases with the depth of the goaf, stabilizing at around 5%. The greater the nitrogen injection amount, the greater the decrease in O_2_ and CO concentrations. Hu Lintao et al.^22^ addressing the limitations of traditional nitrogen injection, such as poor continuity and pipeline resource waste, proposed a new type of “rotary tow-type” continuous and precise nitrogen injection fire suppression equipment. The results showed that CO concentration decreased by 70%, and compared to the 0.38% observed in the underground pipe nitrogen injection process, the extraction area was reduced by 45%. Zhang Lin et al.^23^ studied the impact of nitrogen injection pressure variations on O_2_ concentration changes in the goaf, with results showing that the closer the pressure is to the coal seam gas’s original pressure, the shorter the gas balance time underground. Zhou Xihua et al.^24^ to address fire prevention issues in the goaf, used CFD simulations to study the impact of nitrogen injection parameters on O_2_ concentration in the goaf. The results showed that with a nitrogen injection rate of 500 m^3^/h and an injection depth of 60 m, the natural risk in the goaf could be effectively reduced.

Building on previous research, this study, based on the actual conditions of the 81203 working face at the Baode coal mine, employs Fluent numerical simulation software to construct a goaf model. Through simulations, the effects of varying nitrogen injection locations and volumes on the distribution of the “three zones” of oxidation in the goaf are analyzed, aiming to provide valuable references for enhancing safe production in coal mines.

## 2 Overview of the Working Face

The 81,203 working face at the Baode Coal Mine is situated in the 8# coal seam of the Erpan area. To the east lies the 81,202 working face, and to the west is the 81,204 working face, with surface elevations ranging from 960 to 1084 m. The average thickness of the coal seam is 7.6 m, with an inclination angle varying between 3 and 13°, averaging approximately 6°. The coal seam exhibits high mining feasibility (mining index of 1) and a variation coefficient of 27.5%, indicating its stability. The mining face measures 2812 m in length and 240 m in width. Three tunnels are arranged in the working face: the 81,203 auxiliary transport drift, which is responsible for material transport, personnel access, and air intake for the working face; the 81,203 auxiliary belt conveyor drift, which handles coal transport and air intake, with a transfer machine, crusher, and conveyor belt arranged in this tunnel; and the 81,204 auxiliary transport drift, which is tasked with the return air and the installation of gas drainage pipelines in the goaf.

The mining recovery process is centered on the coal cutting and coal placing procedures, following the basic process of one cut, one place, with parallel operations for mining and placing. The process flow is as follows: coal cutting → move the support → push the front scraper conveyor → coal placing → pull the rear scraper conveyor. The 81,203 working face adopts the “U + L” ventilation system, with the following configuration: the 81,203 auxiliary transport drift serves as the intake air tunnel. The airflow descends through the working face and, after merging with the intake airflow from the 81,203 auxiliary belt conveyor drift at the nearest linking tunnel, flows into the 81,204 auxiliary transport drift. When the working face is 10 to 30 m away from the nearest return air linking tunnel, the next linking tunnel of the 81,203 auxiliary belt conveyor drift is opened to serve as the return air tunnel, and this pattern continues. The ventilation system is shown in Figure 1.

**Figure 1:**
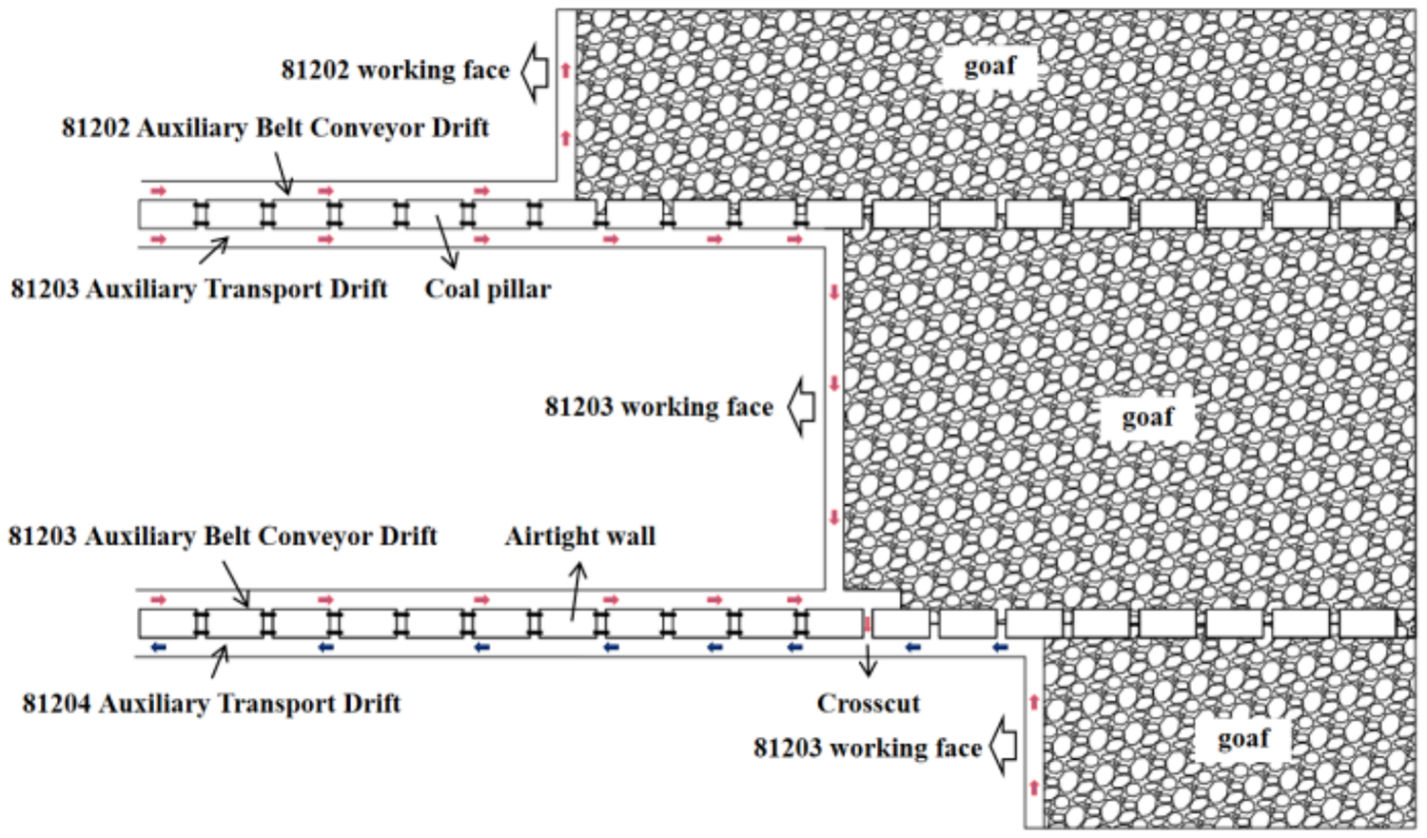
81,203 working face ventilation method.

Since the working face is located in a high gas concentration coal mine, nitrogen injection is mainly used to suppress the spontaneous combustion of residual coal in the goaf. The nitrogen injection pipeline is laid in the 81,203 auxiliary transport drift, and continuous nitrogen injection is recommended. During normal mining recovery, intermittent nitrogen injection can be used; if signs of spontaneous combustion occur, nitrogen should be promptly injected into the goaf to prevent fires until the signs of combustion are eliminated.

## 3 Model Construction and Parameter Settings

### 3.1 Establishment of the Physical Model

Based on the actual conditions of the 81,203 working face of the Baode coal mine, a relatively simple three-dimensional (3D) ventilation system model was established using Space Claim software, as shown in Figure 2. The intersection of the 81,203 auxiliary belt conveyor drift and the working face is set as the origin (O) of the coordinate system. The positive direction of the *X*-axis points to the deep part of the goaf, with the horizontal plane as the XOY plane. The *Y*-axis points from the origin (O) toward the direction of the 81,204 auxiliary transport tunnel, forming an angle of 6° with the working face. The *Z*-axis is determined according to the Right-Handed Coordinate System. The geometric model parameters of the goaf are shown in Table 1. Physical models corresponding to different nitrogen injection hole positions were constructed to reduce errors caused by varying mesh densities.

**Figure 2:**
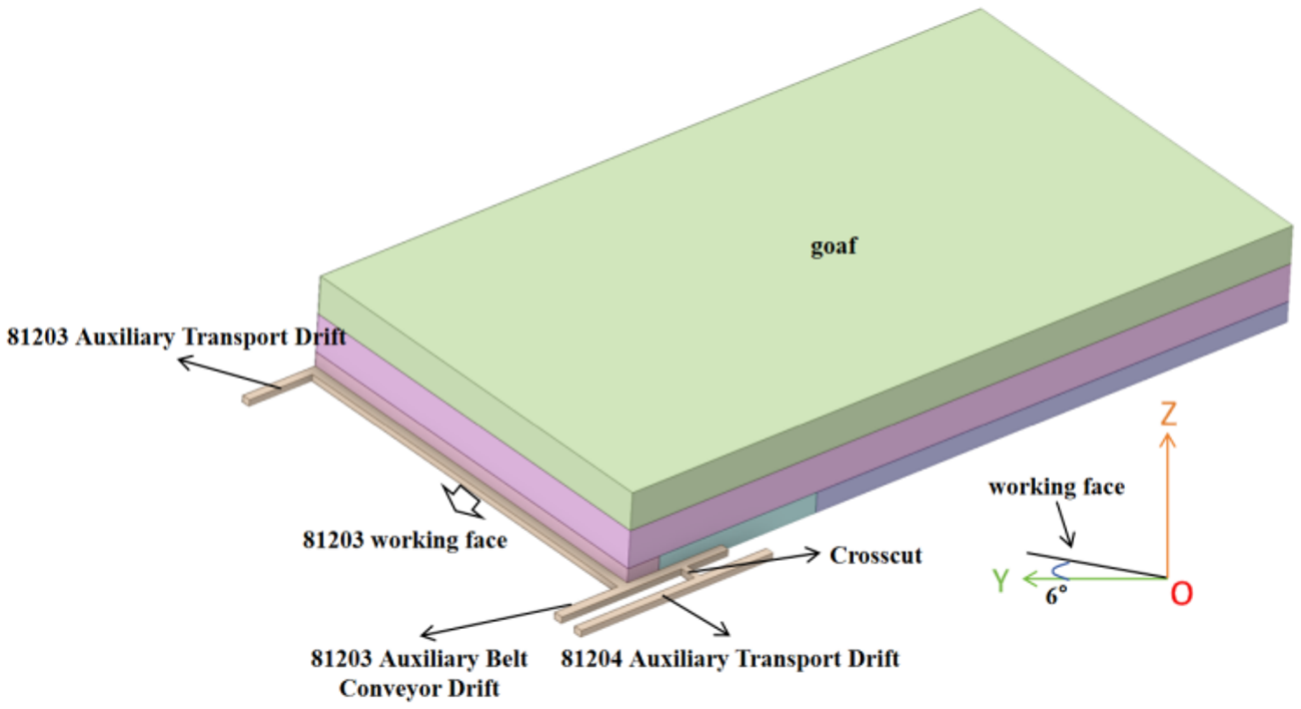
Three-dimensional model of the 81,203 working face ventilation system in the goaf.

**Table 1:** Geometric Parameters of the Model

project	geometric parameters (length, width, and height)
81,203 working face	240 m × 6 m × 3.8 m
81,203 auxiliary transport drift	45 m × 5 m × 3.8 m
81,203 auxiliary belt conveyor drift	76 m × 5 m × 3.8 m
81,204 auxiliary transport drift	76 m × 5 m × 3.8 m
crosscut	10 m × 3.8 m × 3.8 m

### 3.2 Mesh Division of the Physical Model

Following the principles of mesh division, through sensitivity analysis, the most critical zones in the goaf (including various roadways, working faces, and other relatively small areas) were identified, where mesh refinement was implemented. Meanwhile, coarser meshes were maintained in larger areas that have minimal impact on nitrogen injection performance, thereby effectively reducing unnecessary computational complexity. ICEM CFD software was used to mesh all the constructed physical models, ensuring that the mesh quality was ≥0.3. The mesh quality in this study is all above 0.4, meeting the minimum requirement of 0.3.

### 3.3 Boundary Condition Settings

Using Fluent simulation software, the boundary condition at the intake air tunnel entrance is set as Velocity-inlet, and the boundary condition at the return air tunnel outlet is set as Pressure-outlet. The interface between the working face and the goaf is set as Interior, while other boundary conditions are set as Wall. The cell region of the goaf is defined as a porous media zone, and the porosity, viscous resistance coefficient, inertial resistance coefficient, as well as the gas source term and oxygen consumption source term for the goaf are loaded into Fluent software by writing CFD programs.

### 3.4 Solver Parameter Settings

The pressure-based solver type is selected, and a steady-state solution is obtained using an absolute velocity formulation. Due to the coal seam dip angle of 3–13° at the 81203 fully mechanized mining face of Baode coal mine, an acceleration in the negative direction of the *Z*-axis is added and set to −9.8m/s^2^. The pressure–velocity coupling is performed using the SIMPLE algorithm. The momentum equation, turbulence kinetic energy, and turbulence dissipation rate are all discretized using the second-order upwind scheme to enhance the accuracy of the simulation. The turbulence model chosen is the Realizable k-epsilon model,^25^ and the wall function used is the scalable wall function.

## 4 “Three-Zone” Theory of Spontaneous Combustion in the Goaf

### 4.1 Analysis of the “Three-Zone” Distribution of Spontaneous Combustion in the Goaf

In the coal mining process, a significant amount of residual coal remains in the goaf. Based on the difficulty of spontaneous combustion in the goaf, the area can be divided into three zones along the horizontal direction: the heat dissipation zone, the oxidation zone, and the suffocation zone,^26^ as shown in Figure 3. Near the working face, due to ventilation, the hot air is replaced, making it difficult for spontaneous combustion conditions to be met, and the oxygen content remains high, which is why this area is called the oxidation zone. As the distance from the working face increases, the goaf has sufficient oxygen supply, and the heat is hard to dissipate, leading to the conditions for coal self-ignition, resulting in the oxidation zone. Farther from the working face, the oxygen concentration decreases, making it unable to support the combustion conditions for coal self-ignition, hence this area is called the suffocation zone. As the working face continuously advances, the conditions of the residual coal in the goaf also change, so the “three zones” of spontaneous combustion in the goaf are dynamic and constantly changing.

**Figure 3:**
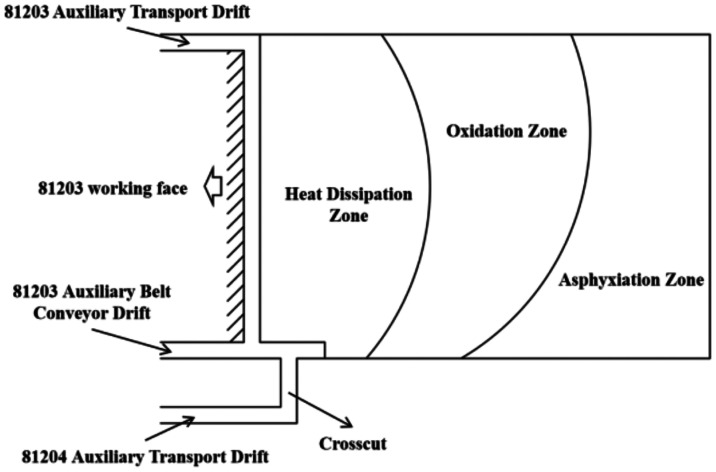
Division of the three zones of coal spontaneous combustion.

### 4.2 Criteria for the Division of the “Three Zones” of Spontaneous Combustion in Goaf Areas

Due to the complex and variable conditions within the goaf, and the limited detection technologies currently available for goaf monitoring, there is no unified standard for the division of the “three zones” of spontaneous combustion. Among the various studies conducted by scholars and experts, the following division criteria are widely recognized.

#### 4.2.1 Oxygen Concentration in the Goaf

The division of the “three zones” of coal spontaneous combustion in the goaf is based on oxygen volume fractions of 18 and 8%. Areas with an oxygen volume fraction greater than 18% are considered the heat dissipation zone, those between 18 and 8% are the oxidation zone, and areas with an oxygen volume fraction less than 8% are classified as the suffocation zone.^27^ This is also the classification criterion adopted in this article.

#### 4.2.2 Heating Rate of Residual Coal in the Goaf

When the temperature rise rate in the goaf reaches *x* ≥ 1 °C/day, it indicates that the coal seam has transitioned from the heat dissipation zone to the oxidation zone. At this point, the oxidation reaction of the coal seam intensifies, causing a rapid temperature increase and releasing a significant amount of heat. When the temperature rise rate drops to *x* ≤ −1 °C/day, the temperature in the goaf begins to decrease, and the oxidation reaction of the coal seam slows down, entering the suffocation zone. At this stage, the oxidation process of the coal seam nearly stops, the temperature decreases, and the risk of coal spontaneous combustion is significantly reduced.^28^

#### 4.2.3 Leakage Intensity in the Goaf

Goaf leakage refers to the infiltration of air into the goaf through various pathways, which affects mine ventilation and safety, leading to issues such as gas accumulation, spontaneous combustion, and air pollution. The primary sources of leakage air include pressure differentials in the ventilation system, poor sealing in the goaf, and seepage through fractures. When the leakage airflow speed in the goaf exceeds 0.24 m/min, it is classified as the heat dissipation zone; when the leakage airflow speed is between 0.1 and 0.24 m/min, it is classified as the oxidation zone; when the leakage airflow speed is less than 0.1 m/min, it is classified as the suffocation zone.^29^

## 5 Simulation Results and Analysis

### 5.1 Simulation Study of Different Nitrogen Injection Locations

To analyze the impact of different nitrogen injection locations on the injection effectiveness and determine the optimal injection location, five nitrogen injection holes were selected at different positions along the 81203 auxiliary transport drift in the goaf. The injection locations were set at distances of 60, 70, 80, 90, and 100m from the working face, with a nitrogen flow rate of 500m^3^/h and a temperature of 293 K. The corresponding results were obtained through Fluent numerical simulations, as shown in Figure 4.

**Figure 4:**
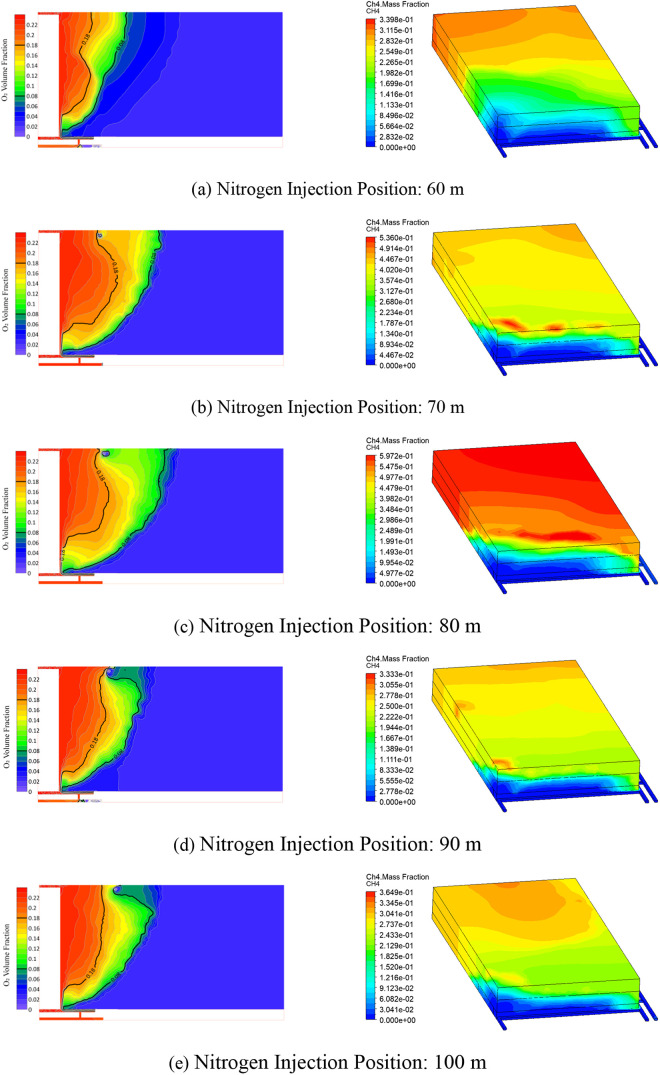
O_2_ and CH_4_ concentration contours at 500 m^3^/h nitrogen injection with different distances. (a, b, c, d, and e) represent nitrogen injection points located 60, 70, 80, 90, and 100 m from the working face, respectively.

The results indicate that as the nitrogen injection hole moves farther from the working face, the width of the oxidation zone in the goaf changes significantly. When the injection hole is positioned between 60 and 80m, the width of the oxidation zone at its widest point increases sharply to approximately 135m. However, as the injection hole moves further away from the working face, the width of the oxidation zone decreases. At a distance of 90m from the working face, the oxidation zone narrows to its minimum value of 77m; but once the injection hole is located beyond 90m, the width of the oxidation zone increases sharply.

Considering the methane content in the goaf, as the injection distance increases, the methane content gradually increases to 59.72% when the injection hole is at 80m. When the injection hole distance is further increased to 90m, the methane content decreases to 33.33%. As the injection distance continues to increase, the methane content increases again.

In conclusion, under the conditions of a nitrogen flow rate of 500m^3^/h and nitrogen injection holes positioned between 60 and 100m, the optimal injection location is found to be at the 81,203 auxiliary transport drift side of the goaf, approximately 90m from the working face. This location effectively narrows the width of the oxidation zone, thereby optimizing ventilation and fire suppression effectiveness.

### 5.2 Simulation Study on Different Nitrogen Injection Amounts

The nitrogen injection port was set on the 81,203 auxiliary transport drift side of the goaf, 90 m away from the working face, with the nitrogen flow rate increasing from 100 to 900 m^3^/h in increments of 100 m^3^/h. The results of the numerical simulation are shown in Figure 5.

**Figure 5:**
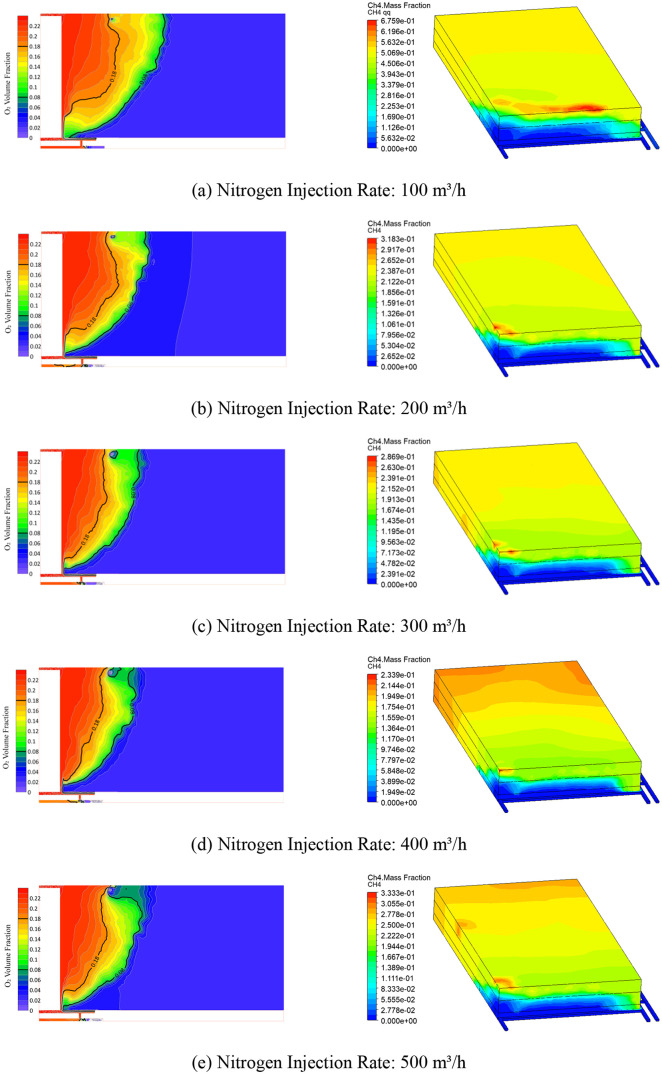

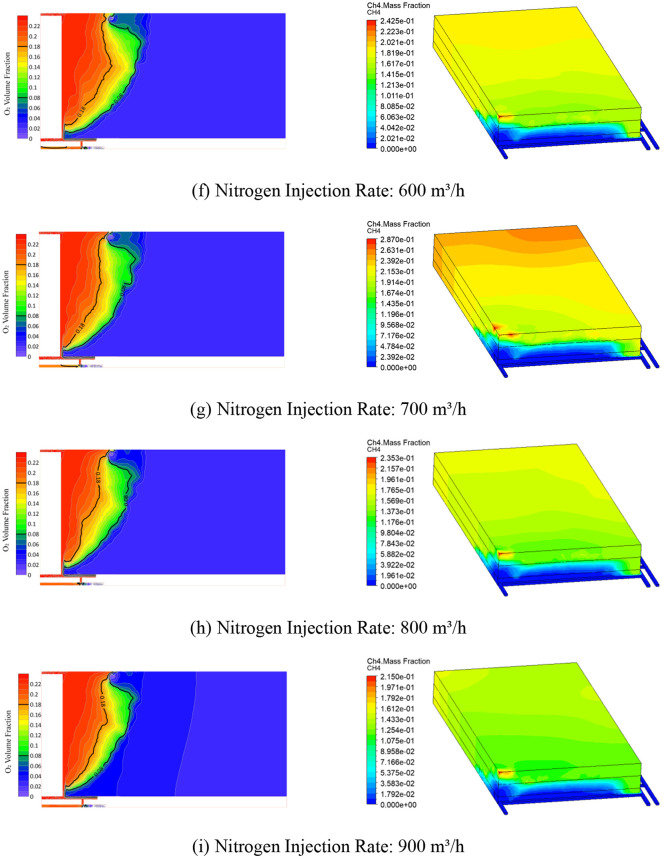
O_2_ and CH_4_ concentration contours at 90 m injection distance with different nitrogen injection rates. (a, b, c, d, e, f, g, h, and i) represent nitrogen injection rates of 100, 200, 300, 400, 500, 600, 700, 800, and 900 m^3^/h, respectively.

The results indicate that when the nitrogen injection amount is low, the width of the oxidation zone is larger. As the nitrogen injection amount increases, the width of the oxidation zone rapidly decreases, until the injection rate reaches 400 m^3^/h, at which point the width of the oxidation zone slightly increases. Further increasing the injection amount causes the width of the oxidation zone to stabilize and gradually decrease.

Considering the methane concentration, when the nitrogen injection amount is 400 m^3^/h, the maximum methane concentration in the goaf is 23.39%, which is lower compared to the maximum methane concentrations under the conditions of 300 and 500 m^3^/h injection rates.

In summary, the optimal nitrogen injection location for the 81203 working face of the Baode coal mine is about 90 m from the working face, with the optimal nitrogen injection rate being 400 m^3^/h. As the width of the oxidation zone dynamically changes with the advancement of the working face, the nitrogen injection hole position should be adjusted in a timely manner to avoid resource waste and ensure the safe production of the coal mine.

## 6 Conclusions

(1) Based on the parameters of the 81203 working face at Baode coal mine, a physical model was established to study the distribution of spontaneous combustion “three zones” in the goaf under nitrogen injection conditions. The study identified the optimal nitrogen injection location and flow rate, providing scientific guidance for coal mine safety production and engineering practices. These findings contribute to the optimization of nitrogen injection fire prevention schemes and enhancement of fire suppression effectiveness.
(2) Fluent simulations were employed to analyze the distribution of the “three zones” under different nitrogen injection locations while maintaining a constant injection rate. The results indicate that at a nitrogen injection rate of 500 m^3^/h, the width of the oxidation zone initially increases, then decreases, and subsequently rises again as the injection point moves farther from the working face. The minimum oxidation zone width (77 m) occurs at a distance of 90 m from the working face, making this location the optimal injection point under the given conditions.
(3) With the nitrogen injection point fixed at 90 m from the working face, increasing the injection rate leads to a gradual reduction in the oxidation zone width. However, when the injection rate reaches 400 m^3^/h, a temporary rebound in the oxidation zone width is observed before further decline and stabilization. Considering economic efficiency, 400 m^3^/h is determined to be the optimal injection rate, effectively mitigating the risk of coal spontaneous combustion while minimizing unnecessary nitrogen consumption, thereby improving cost-effectiveness.
(4) Numerical simulation offers significant advantages in studying the influence of nitrogen injection on the “three zones”, including high visualization, low cost, and strong controllability. This approach generates intuitive visual representations of the dynamic changes in the “three zones”, addressing the limitations of traditional theoretical calculations. Moreover, it serves as a viable alternative to costly and complex field experiments, substantially reducing research expenses. The flexibility to adjust variables such as injection rate, location, and ventilation conditions enhances the precision and optimization potential of the study, ultimately leading to the development of optimal nitrogen injection strategies and improving the scientific and practical aspects of coal mine safety production.

## Data Availability

The data supporting the findings of this study are not available at this stage, as the research is still ongoing and forms part of a broader investigation. To ensure the integrity and continuity of the research, data sharing is not possible at this time.
